# A Serial Multi-Scale Feature Fusion and Enhancement Network for Amur Tiger Re-Identification

**DOI:** 10.3390/ani14071106

**Published:** 2024-04-04

**Authors:** Nuo Xu, Zhibin Ma, Yi Xia, Yanqi Dong, Jiali Zi, Delong Xu, Fu Xu, Xiaohui Su, Haiyan Zhang, Feixiang Chen

**Affiliations:** 1 School of Information Science and Technology, Beijing Forestry University, Beijing 100083, China; xu993790@bjfu.edu.cn (N.X.); mmazb@bjfu.edu.cn (Z.M.); xiayi@bjfu.edu.cn (Y.X.); yanqidong@bjfu.edu.cn (Y.D.); jializi@bjfu.edu.cn (J.Z.); xudelong@bjfu.edu.cn (D.X.); xufu@bjfu.edu.cn (F.X.); suxhui@bjfu.edu.cn (X.S.); zhyzml@bjfu.edu.cn (H.Z.); 2 Engineering Research Center for Forestry-Oriented Intelligent Information Processing, National Forestry and Grassland Administration, Beijing 100083, China

**Keywords:** Amur tiger, intelligent recognition, deep learning, double branch structure, feature pyramid, attention mechanism

## Abstract

**Simple Summary:**

The Amur tiger is an endangered species in the world, and effective statistics on its individuals and population through re-identification will contribute to ecological diversity investigation and assessment. Due to the fact that the fur texture features of the Amur tiger contain genetic information, the main method of identifying Amur tigers is to distinguish their fur and facial features. In summary, this paper proposes a serial multi-scale feature fusion and enhancement network for Amur tiger re-identification, and designs a global inverted pyramid multi-scale feature fusion module and a local dual-domain attention feature enhancement module. We aim to enhance the learning of fine-grained features and differences in fur texture by better fusing and enhancing global and local features. Our proposed network and module have achieved excellent results on the public dataset of the ATRW.

**Abstract:**

The Amur tiger is an important endangered species in the world, and its re-identification (re-ID) plays an important role in regional biodiversity assessment and wildlife resource statistics. This paper focuses on the task of Amur tiger re-ID based on visible light images from screenshots of surveillance videos or camera traps, aiming to solve the problem of low accuracy caused by camera perspective, noisy background noise, changes in motion posture, and deformation of Amur tiger body patterns during the re-ID process. To overcome this challenge, we propose a serial multi-scale feature fusion and enhancement re-ID network of Amur tiger for this task, in which global and local branches are constructed. Specifically, we design a global inverted pyramid multi-scale feature fusion method in the global branch to effectively fuse multi-scale global features and achieve high-level, fine-grained, and deep semantic feature preservation. We also design a local dual-domain attention feature enhancement method in the local branch, further enhancing local feature extraction and fusion by dividing local feature blocks. Based on the above model structure, we evaluated the effectiveness and feasibility of the model on the public dataset of the Amur Tiger Re-identification in the Wild (ATRW), and achieved good results on mAP, Rank-1, and Rank-5, demonstrating a certain competitiveness. In addition, since our proposed model does not require the introduction of additional expensive annotation information and does not incorporate other pre-training modules, it has important advantages such as strong transferability and simple training.

## 1. Introduction

Widespread distribution, low population density, unpredictable behavior patterns, and sensitivity to interference of wildlife pose significant challenges to monitoring work for some animal species. Traditional wildlife investigation techniques mainly include manual investigation, line sampling, collar tracking, and acoustic tracking using sound recording instruments [1,2]. However, each of these methods has certain disadvantages, so scientists strive to improve them. The Amur tiger, also known as the Siberian Tiger, is one of the subspecies of tigers. The Amur tiger is mainly distributed in the northeastern region of Asia and is listed as an endangered species in the Red List of Threatened Species by the World Conservation Union. There are only just over 500 Amur tigers left in the world, so it is crucial to strengthen the protection of the Amur tiger [3]. Moreover, the survival and reproduction of species populations are closely related to regional biodiversity and ecosystem functional integrity [4]. Therefore, re-evaluating the Amur tiger and its prey resources in natural environments such as nature reserves and national parks can help to statistically analyze the situation of Amur tiger resources and provide data reference for the next step of protection work [5,6]. At present, the most commonly used method for the re-ID of wild animals is manual discrimination. After receiving professional knowledge and training, wildlife protection professionals need to screen and distinguish a large amount of image data based on the fur pattern characteristics of the abdomen, head, neck, and other parts of the Amur tiger [7,8]. To reduce errors, it is necessary for multiple people to simultaneously identify and verify the recognition results, which requires a large workload, high cost, and low efficiency.

With the continuous development and application of machine learning and deep learning technologies, machine learning algorithms and emerging deep learning models, such as Linear Clustering [9], Classification [10,11], Detection [12,13], and Generative Adversarial Networks [14], are gradually being applied to the intelligent monitoring and protection of wildlife. Research on the intelligent recognition of wildlife mainly focuses on issues such as wildlife re-ID, species classification, population counting, and attribute recognition [15]. In the process of wildlife re-ID, the application of computer vision-related technologies can greatly improve recognition efficiency and accuracy. Research in this area has gradually become popular. Currently, the main methods used are clustering algorithms based on image hotspots [9] and convolutional neural network models based on VGG [16], AlexNet [17], and ResNet [18]. These methods have been improved in optimizing feature extraction, feature fusion, and incorporating prior knowledge of pose. Zheng et al. proposed a Transformer network structure with cross-attention block (CAB) and local awareness (CATLA Transformer) [19], which captures global information of an animal body’s surface and local feature differences in fur, color, texture, or face, and fuse global features and local features through CATLA Transformer. Zhang et al. proposed using texture features as global and local features for re-ID, and proposed a pyramid feature fusion model method to extract features from both local and global perspectives, effectively matching entities [20]. Li et al. proposed an Amur tiger re-ID method, which introduces precise pose parts with deep neural networks to handle the large pose variation of tigers [3]. Liu et al. proposed a Partial Pose Guided Network (PPGNet), which uses local image features based on pose data to drive the network to extract features from the original image, and applies it to an Amur tiger re-ID system based on automatic detection and Amur tiger pose estimation [21]. He et al. proposed a Multi-pose Feature Fusion Network (MPFNet), which constructs three pose modules: standing, sitting, and lying. In each module, two parallel branches are used to extract global and local features for effective feature extraction. Finally, the features are fused [22].

There are also some very advanced studies in the field of person re-identification similar to the Amur tiger re-ID. Sun et al. proposed a Part-based Convolutional Baseline (PCB) framework and an inter-block combination method with uniform partitioning to effectively extract part-level features, and by Refined Part Pooling (RPP), closer parts are allocated together to improve the within-part consistency of parts [23]. Sun et al. considered the problem of partial re-ID and proposed a Visibility-aware Part Model (VPM). Through self-supervised learning, the model perceives the features within the visible region, extracts regional features, and compares two images within their shared regions to suppress noise in unshared regions. It better extracts fine-grained features of the image and reduces image misalignment [24]. Liu et al. proposed a multi-scale Feature Enhancement (MFE) Re-ID model and a Feature Preserving Generative Adversarial Network (FPGAN). In the MFE, the semantic feature maps of the person’s body are segmented, and then multi-scale feature extraction and enhancement are performed on the person’s body region. In the FPGAN, the source domain is transferred to the target domain in an unsupervised manner, maximizing the preservation of personal information integrity [25].

In current research, there are issues such as the need for prior knowledge and the complexity of training large models. Although some models have been verified to have excellent average accuracy and other indicators, and the effectiveness of model improvement has been verified through ablation experiments, a large amount of reliance on prior knowledge leads to poor model transferability, requiring staff with expertise in wildlife to perform a large amount of dataset labeling and processing work in the early stages before retraining the model, which has low feasibility in practical production applications. The networks with four, six, or more branches, or which require data preprocessing through instance segmentation models before being fed into the re-ID model, are too complex and have problems such as large model size and a need for complex training. Therefore, this paper proposes a serial multi-scale feature fusion and enhancement re-ID network of Amur tigers with global inverted pyramid multi-scale feature fusion and local dual-domain attention feature enhancement for the re-ID of Amur tiger images. Combining the re-ID methods of the Amur tiger and fine-grained task properties, the Path Aggregation Network (PANet) [26] feature fusion idea is introduced. A bottom-up unidirectional feature fusion method is proposed, which uses an inverted pyramid structure for feature fusion. This helps to better integrate high-level features with large receptive fields and rich semantic information while preserving multi-scale features. We propose a local dual-domain attention feature enhancement method that is serially connected with the global branch to enhance local feature extraction and fusion. Our goal is not to go beyond the SOTA model used for re-ID, but to propose an end-to-end model that is more suitable for removing animal pose prior knowledge and other additional attribute information, and has good transferability and re-ID performance. Our core contributions are as follows:

- We integrate and propose a lightweight, efficient, end-to-end network for the re-ID task of the Amur tiger, which does not require the introduction of prior knowledge such as posture. It can be quickly and conveniently used for the re-ID task of other large mammals. The specific network innovation and design are as follows.
- In order to better extract and integrate the global information of the high-level and low-level layers of the Amur tiger, we propose a multi-scale feature fusion method of the global inverted pyramid. We introduce the ideas of Feature Pyramid Network (FPN) [27] and PANet into the global branch of the model for the task of wildlife re-ID. Improving the top-down connection method of traditional feature pyramid models will greatly compress the problem of key deep semantic information [28].
- In order to deepen the feature extraction of various parts of the Amur tiger and extract fine-grained features such as body fur texture, we introduce a serial local branch network and design an attention module and output feature fusion method in the local branch.

## 2. Materials and Methods

This section mainly introduces the dataset we use, the basic process of Amur tiger re-ID, and the structure and details of our proposed Amur tiger re-ID network.

### 2.1. Dataset

**Figure 1:**
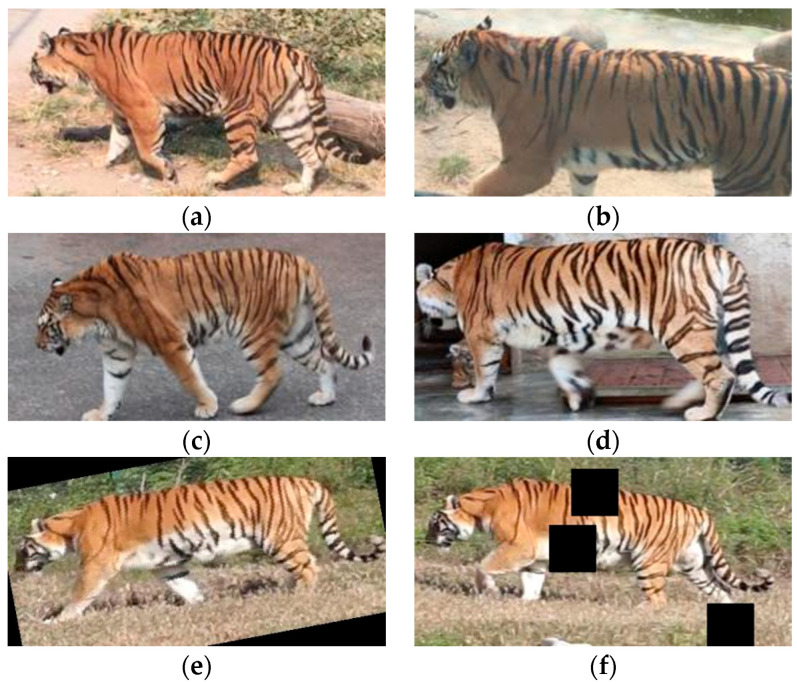
(**a**–**d**) are the original images of the public dataset of the ATRW, and (**e**,**f**) are examples of data-enhanced images.

To validate the effectiveness of the proposed method and model, we conducted training, testing, and evaluation using the public dataset of the ATRW [3]. The ATRW dataset is a dataset jointly released by Shanghai Jiao Tong University and Intel Laboratories with the assistance of the World Wildlife Fund International (WWF) in 2019 for the detection, joint estimation, and re-ID tasks of the Amur tiger. The authors of this dataset collected over 8000 video clips of 92 tigers from approximately 10 zoos in China to create the ATRW dataset. After data organization and classification, the training dataset for the re-ID task contains 107 tiger entities, totaling 1887 images. The test dataset contains 47 tiger entities, with a total of 701 images. To enhance the robustness of the model and increase the number of training samples, we performed random rotation and random occlusion enhancement on some images in the training dataset, and set the image size H × W to 256 × 512 for better results [21]. We reduced the influence of external factors such as shooting angle and shooting position, and randomly divided the data into the training set Train and validation set Val in a 7:3 ratio (Table 1). The train set we use has an average of 18 training images per entity after data augmentation, with at least 9 training images for each entity (Figure 1).

### 2.2. Methods

#### 2.2.1. Serial Multi-Scale Feature Fusion and Enhancement re-ID Network of Amur Tiger

**Figure 2:**
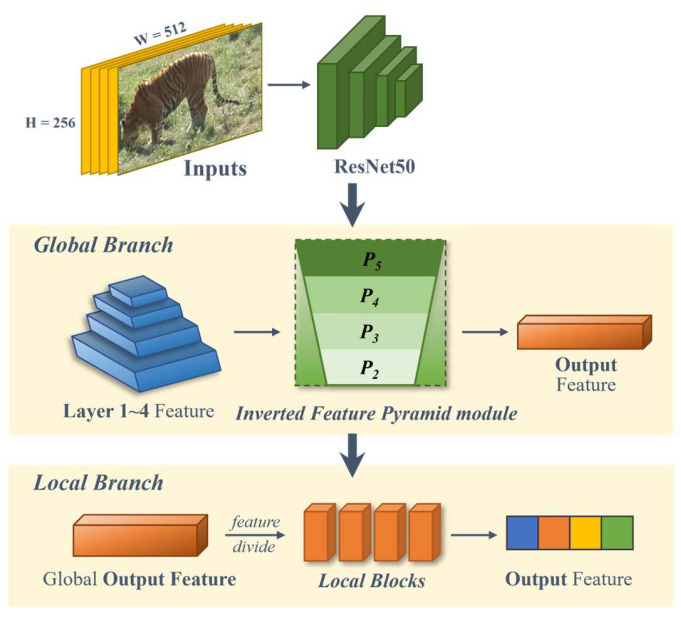
Our proposed Amur tiger re-ID network structure.

In this paper, we propose a network aimed at completing the task of Amur tiger re-ID, which is a serial multi-scale feature fusion and enhancement re-ID network of the Amur tiger (Figure 2). The network is mainly divided into two parts: a global branch and a local branch, which are combined to achieve the final effect. ResNet50 is a convolutional neural network with a depth of 50 layers, which has excellent classification performance on ImageNet [18]. Its pre-trained model was trained on ImageNet with over 1 million images. Therefore, our proposed re-ID network applies the backbone ResNet50 and removes the last down-sampling layer of ResNet50 to retain a larger scale [29]. On the basis of the backbone, a dual-branch structure is constructed based on the feature tensor obtained from ResNet50 layer 4 to achieve the localization of the position of the Amur tiger in the image and fine-grained re-ID requirement. Our proposed dual branches are connected in a serial manner, and the feature tensors obtained from the global branch output are sent to the local branch for the next step of feature extraction and fusion (the details of the global and local branches are in Section 2.2.2 and Section 2.2.3). Finally, the feature tensors *F_Global_* ∈ R^2048×1^ and *F_Local_* ∈ R^2048×1^ for global and local branch outputs are obtained, which are concatenated and sent to the classifier layer. Through the linear layer, they are expanded to the corresponding number of categories.

#### 2.2.2. Global Inverted Pyramid Multi-Scale Feature Fusion Method

Taking inspiration from the FPN model and the PANet model, we propose an Inverted Feature Pyramid Module (IFPM) for global multi-scale feature fusion. We attempt to guide the fusion of multi-scale features from coarse to fine and from low-level to high-level, reducing the compression of deep semantic information and maximizing the retention of high-level features, in order to construct a multi-scale feature pyramid dominated by deep semantic information on the global feature branch.

**Figure 3:**
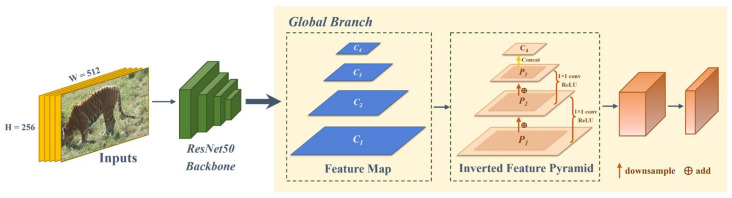
Principle of global inverted pyramid multi-scale feature fusion method.

As shown in the yellow part of Figure 3, we propose a reverse feature fusion path in the global branch. From bottom to top, we use the features extracted from layer 1 to layer 4 in the backbone as input feature maps of the global inverted pyramid multi-scale feature fusion module. Finally, we obtain the output feature map.

Specifically, we define *C* ∈ R^C×H×W^ to represent the features output by each layer of ResNet50, where H × W corresponds to the spatial dimensions of the feature map, and C denotes the number of channels. In the multi-scale feature fusion module of the global inverted pyramid, we designed a multi-scale feature fusion connection strategy. We utilize the features *C*_1_ ∈ R^256×32×64^, *C*_2_ ∈ R^512×16×32^, *C*_3_ ∈ R^1024×8×16^, and *C*_4_ ∈ R^2048×8×16^ extracted from layer 1 to layer 4 in ResNet50 as input features. Starting from *C*_1_, *C*_1_ passes through the down-sampling layer based on the H × W of *C*_2_ to obtain *P*_1_. After adding *P*_1_ to *C*_2_, the next step of feature fusion and ReLU is performed to obtain *P*_2_. Then, based on the H × W dimension of the upper layer of the inverted pyramid, *C*_3_ and *C*_4_ are sequentially subjected to down-sampling, convolutional feature extraction, fusion, concatenation, and ReLU activation to complete the feature connection and fusion at each stage. By continuously mapping from low-level features to high-level features, *P*_1_ ∈ R^512×16×32^, *P*_2_ ∈ R^1024×8×16^, and *P*_3_ ∈ R^2048×8×16^ are obtained. *P*_3_ and *C*_4_ are connected in parallel and average pooling is performed to obtain the complete global multi-scale feature F_Global_ ∈ R^2048×1×1^, maximizing the preservation of deep semantic information and better fitting the fine-grained and deep semantic features of the Amur tiger for re-ID.

#### 2.2.3. Local Dual-Domain Attention Feature Enhancement Method

**Figure 4:**
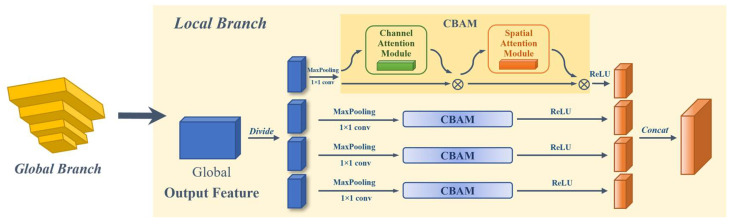
Principle of local dual-domain attention feature enhancement method.

**Figure 5:**
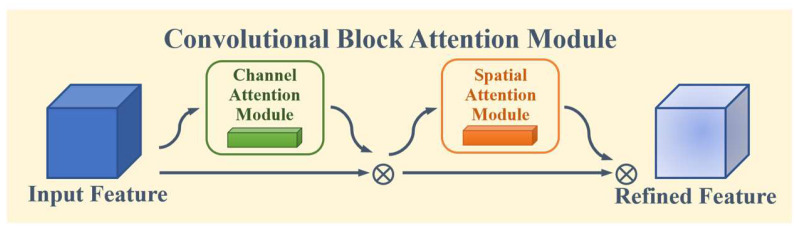
Principle of Convolutional Block Attention Module.

We propose a Local Attention Enhancement Module (LAEM) based on the Convolutional Block Attention Module (CBAM) [30] to enhance the feature extraction performance of multiple local blocks in local branches (Figure 4). CBAM is an attention mechanism module used to enhance the performance of convolutional neural networks, which improves the model’s perception ability by introducing the mixed attention of channel attention and spatial attention (Figure 5). Channel attention helps to enhance the feature representation of different channels, while spatial attention helps to extract key information at different positions in space [30].

The local branch of this network is different from the global branch in fusing features at different scales. The local branch further strengthens and extracts features in different ranges through horizontal blocking, which helps to extract and optimize local details such as the texture and stripes of Amur tiger fur, and improves the accuracy of re-ID.

**Figure 6:**
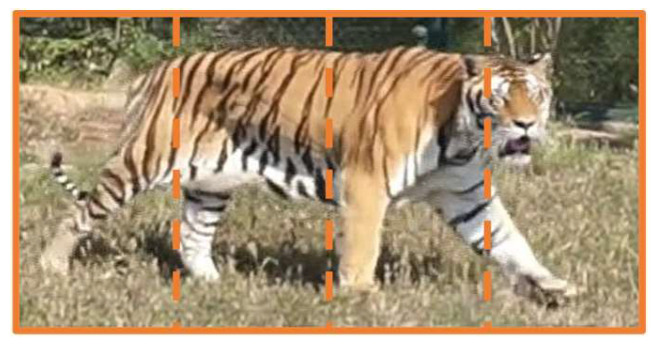
The method of Amur tiger feature map segmentation.

We divide the global feature obtained from the inverted pyramid module of the global branch into 4 blocks from left to right, each block being a local feature *block* ∈ R^2048×8×2^ (Figure 6). In the local branch, we perform adaptive max pooling and 1 × 1 convolution operations on each local block feature to obtain a reduced local feature representation to 512. Then, we feed each reduced feature block into the CBAM. Channel and spatial attention feature enhancements are applied to the local block feature, dual-domain feature representation is enhanced, and important features such as local stripes after block segmentation are enhanced to obtain {*L*_1,_ *L*_2_, *L*_3_, *L*_4_} ∈ R^512×1×1^. Finally, the four feature blocks obtained through attention enhancement were activated using the activation function ReLU, and then concatenated to obtain the final feature output *F_Local_* ∈ R^2048×1×1^ of the local branch.

### 2.3. Training and Reasoning

During the training process, the training dataset of Amur tiger images is input into our proposed serial multi-scale feature fusion and enhancement re-ID network of the Amur tiger. Through global and local branches, feature extraction and classification tasks are carried out through the global inverted pyramid multi-scale feature fusion module and local dual-domain attention feature enhancement module. Finally, the classifier layer is applied for re-ID.

#### 2.3.1. Loss Function

In probability statistics, entropy is a measure of the invariance of random variables. Cross entropy can measure the degree of similarity and difference between two distributions and is often used in image multi-classification and other problems.

We employ the Cross-Entropy Loss, a commonly used logarithmic loss function for multi-class classification problems, to regulate and optimize the training process of our network. The formula is as follows:

(1) $$L_{i}=-\sum_{C=1}^{K}y_{ic}log\left(p_{ic}\right)\;$$

#### 2.3.2. Inference

**Figure 7:**
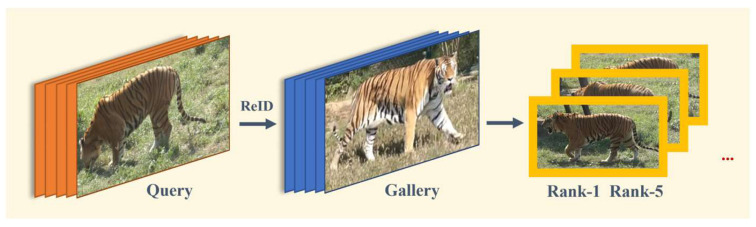
The inference process for Amur tiger re-ID.

During the inference process, when wildlife conservation workers obtain a set of images from camera traps or surveillance videos, we can select any image as the Query and other images as the Gallery. Then, we input the Query and Gallery into the trained network. After feature extraction and re-ID, we can obtain the similarity ranking of all images in the Gallery compared to the Query. After descending sorting, we obtain the most similar entity image of the Amur tiger extracted by the Query in the Gallery. At this time, we can determine the probability from high to low that it is the same as the Amur tiger entity in the Query image (Figure 7).

## 3. Results and Analysis

This section mainly introduces the experimental setup, evaluation methods, and specific experimental results.

### 3.1. Experimental Setup

Our proposed model is implemented in Cuda using the PyTorch framework. The computer and code environments in which we conducted the experiment were configured with PyTorch, Python 3.8, and Cuda11.3, and the entire training and testing process was conducted on a server configured with an NVIDIA GeForce RTX 3090 GPU. The training and testing sets used in the experiment are shown in Table 1. During the model training phase, we use SGD and set the momentum to 0.9, basic learning rate to 0.002, weight decay to 0.0005, and batch size to 16. The learning rate of the classifier layer is set to 0.02, and the learning rate is decreased by a factor of 10 at epoch 100. The dataset was randomly erased and underwent a total of 150 epochs of training.

### 3.2. Evaluation

The testing process of this task involves extracting an image from the Query dataset of the test set and calculating the Euclidean distance between this image and all images in the Gallery except for this image. The Euclidean distance calculation formula is as follows:

(2) $$Distance=sort\left(\left(A-B\right)\cdot \left(A-B\right)\right)$$

Sort in descending order based on the calculation results to determine whether the image extracted by Query and other images in the Gallery are entities with the same ID label:

(3) $$F\left(l_{image},l_{gallery}\right)\;≜\left\{\begin{array}{c} 1\;\;if\;l_{image}=l_{gallery}\\ 0\;\;if\;l_{image}\neq l_{gallery} \end{array}\right.\;$$

The task of Amur tiger re-ID is similar to the sub-task of person re-ID in image retrieval, so the same testing methods and evaluation indicators, such as the CMC curve and mAP, can be used. This paper uses three indicators for re-ID evaluation: Rank-1 Accuracy, Rank-5 Accuracy, and mean average precision (mAP). Rank-1 is the probability of the first image being retrieved hitting, and Rank-5 is the probability of the first five images being retrieved hitting. Rank-1 can be explained using the formula shown in (4), and the calculation method for Rank-5 is similar to this:

(4) $$Rank-1=\frac{1}{\left\|Q\right\|}\underset{q\in Q}{\sum }F\left(l_{image}^{q},l_{gallery}\right)\;$$

mAP reflects the degree to which real images rank higher in sorting, and compared to Rank-1, Rank-5, etc., it can more comprehensively measure the effectiveness of re-ID. Therefore, this indicator is also used as the primary evaluation indicator in this paper.

### 3.3. Compared with Other Advanced Methods

#### 3.3.1. Comparison with Improved Methods Based on ResNet50

**Figure 8:**
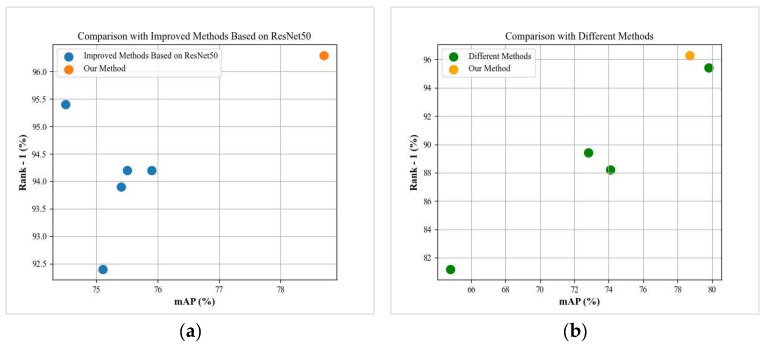
(**a**,**b**) shows the comparison results of our proposed model experimental indicators with other advanced models.

**Figure 9:**
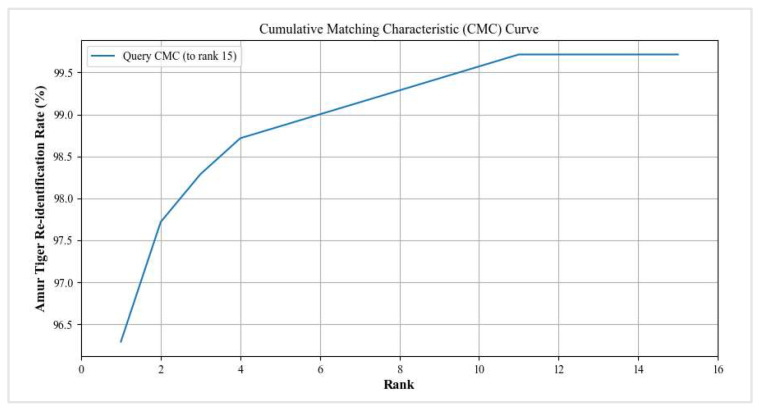
The CMC curve of our Amur tiger re-ID model.

Our proposed method has achieved good results on the public dataset of ATRW (Table 2). Compared with the comparative experimental results of the advanced person re-ID model based on ResNet50, our proposed model has achieved better results and significant improvements (Figure 8 and Figure 9).

#### 3.3.2. Comparison with Different Improvement Methods

**Figure 10:**
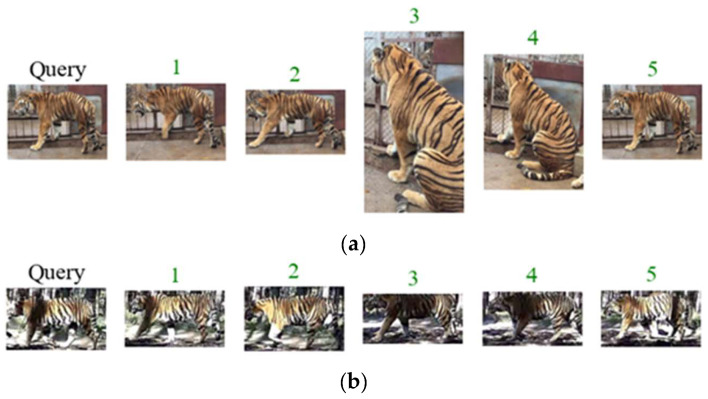
(**a**,**b**) are examples of the results of applying our proposed network for Amur tiger re-ID. The number above the image shows the similarity ranking result, and the green color shows correct re-ID.

The model we propose is an end-to-end re-ID model that does not require additional prior knowledge. It has good transferability and is a significant improvement compared to Aligned-reID, which also combines global and local features. However, compared with models PPbM-a, PPbM-b, and MPFNet, which incorporate or pre-train pose estimation modules, it can still achieve good re-ID performance, with some indicators improved (Table 3). We randomly selected two Query images for Amur tiger re-ID as examples (Figure 10).

### 3.4. Ablation Experiment

To verify the effectiveness of our proposed method and module, we conducted ablation experiments on each part. This model chooses the classic feature extraction and classification model ResNet50 as the backbone. Firstly, we experimentally verify the testing performance of only the backbone. Then, we separately verify the effectiveness of adding the proposed global inverted pyramid multi-scale feature fusion module and local feature enhancement module. The following is a detailed description and explanation.

#### 3.4.1. Effectiveness of the Global Inverted Pyramid Multi-Scale Feature Fusion Module and the Local Dual-Domain Attention Feature Enhancement Module

Due to the fact that our proposed global inverted pyramid multi-scale feature fusion module is constructed based on the ideas of FPN and PANet, we compared the experimental results of models that only used ResNet50 and introduced FPN and PANet based on ResNet50 (Table 4). The introduction of FPN in the backbone resulted in a 7.7% decrease in mAP compared to only using the backbone, while our proposed IFPM method resulted in 76.1% mAP, 96.3% Rank-1, and 98.9% Rank-5. Compared to the model introducing FPN, mAP improved by 9.4%, while Rank-1 and Rank-5 improved by 4.6% and 1.3%, demonstrating significant advantages in these three indicators. This result proves that, as expected by our analysis, adopting deep semantic features to fuse global features in an inverted pyramid shape is more suitable for the re-ID task of the Amur tiger.

The experimental results of fusing IFPM and LAEM dual modules showed a 2.6% improvement in mAP compared to the model containing only IFPM modules, while the results of Rank-1 and Rank-5 remained unchanged, demonstrating the effectiveness of the LAEM module and the serial combination of IFPM and LAEM (Table 5).

#### 3.4.2. Effectiveness of CBAM in Local Dual-Domain Attention Feature Enhancement Module

Due to the application of CBAM in our proposed local dual-domain attention feature enhancement module, we compared the experimental results of models using other robust and effective attention modules to demonstrate the optimal performance of CBAM in this module.

The experimental results of introducing Squeeze-and-Excitation Network (SENet) and Efficient Channel Attention (ECA) into our improved model, which incorporates IFPM, showed a 1.7% improvement in mAP compared to the backbone, while Rank-1 and Rank-5 increased by 1.3% and 2%, respectively (Table 6). Our proposed module incorporating CBAM improved mAP by 2.6% compared to the model incorporating SENet, while Rank-1 and Rank-5 improved by 1.6% and 0.5%, respectively. Compared to the model incorporating ECA, mAP improved by 2.6%, while Rank-1 and Rank-5 improved by 0.9% and 0.5%, respectively.

## 4. Discussion

The Amur tiger may have the problem of occupying a relatively large position and having a relatively complex image background in the photos captured by camera traps [34]. This is because photo shooting is triggered only when the wild animals are relatively close to the infrared camera and the infrared sensor senses their temperature [34,35]. Moreover, due to the complex forest environment, the Amur tiger has a narrow path and a larger target. Therefore, we propose a new serial multi-scale feature fusion and enhancement re-ID network of Amur tiger, which extracts and learns to input Amur tiger features in a global and local branch serial manner. We also propose a global inverted pyramid multi-scale feature fusion method and a local dual-domain attention feature enhancement method to learn Amur tiger images at multiple scales, more adaptable to this re-ID task. In the model validation stage, we applied the Amur tiger re-ID dataset of the ATRW for experimental verification. The experimental results showed that our proposed model still has good performance without introducing other prior knowledge and complex labeling, and the mAP and hit rate have been improved. In addition to the Amur tiger, our proposed network is applicable to other large quadruped animals through retraining. It can be structurally adjusted according to specific animal species and task details, without the need to introduce other prior knowledge, reducing the cost of early labeling and other inputs, and has a certain degree of universality and transferability.

In summary, since our constructed model requires vertical partitioning of extracted features in the horizontal direction, it is effective in identifying large quadruped mammals that are mostly identified by body surfaces, such as snow leopard re-ID and leopard species classification. However, we have not yet conducted further validation and model fine-tuning on many other large quadruped animal datasets, and if we apply datasets of upright animals such as monkeys, there may be issues with poor performance. Because our proposed method requires local partitioning in the horizontal direction, and for such animals, the key complete features may be segmented, resulting in the inability to learn important information. Currently, however, a suitable dataset for comparative experiments remains unavailable. This is the limitation and problem that this paper aims to address, and further in-depth research is still needed. In the future, we will strive to create datasets and complete research and comparative experiments on model transfer. In addition, this paper is conducted on a public dataset where each entity has an average of 14.5 and at least 8 training images prior to data augmentation. However, in real life, there may be small and uneven sample sizes in the dataset we obtain in the wild or in surveillance videos, which are also issues that we need to address in the future.

## 5. Conclusions

The re-ID and counting of Amur tigers play an important role in studying and analyzing the entity quantity and distribution of various populations, biodiversity, and individual tracking of wild animals. Therefore, improving the mAP, Rank-1, and Rank-5 of Amur tiger re-ID has enormous ecological significance and value. In order to improve the accuracy and efficiency of the Amur tiger re-ID, we propose a serial multi-scale feature fusion and enhancement re-ID network of the Amur tiger. A global inverted pyramid multi-scale feature fusion method and a local dual-domain attention feature enhancement method were designed for this network. Our proposed network and method have the advantages of no prior knowledge, high efficiency, and end-to-end functionality. Through experiments on the public dataset ATRW of Amur tiger and comparative experiments with other methods, it has been proven that this method has good performance and will help improve the re-ID performance of Amur tiger.

## Figures and Tables

**Table 1:** Training and Testing dataset used in the experiment.

Train Dataset	Amur Tiger Entities	Amur Tigers	Original Images
Train + Val	107	75	1552 + 35
**T** **est Dataset**	**Amur Tiger Entities**	**Amur Tiger** **s**	**Original Images**
Query	47	42	701
Gallery	47	42	701

**Table 2:** Comparison with ResNet50 based method on ATRW test dataset.

Method	mAP	Rank-1	Rank-5
PCB [23]	74.5%	95.4%	98.7%
ResNet50 + Triplet Loss [29]	75.1%	92.4%	99.1%
ResNet50 + IBN [31]	75.4%	93.9%	98.4%
ResNet50 + Lifted Loss [32]	75.5%	94.2%	98.7%
ResNet50 + Circle Loss [33]	75.9%	94.2%	98.9%
Ours	78.7%	96.3%	98.9%

**Table 3:** Comparison with different methods on the ATRW test dataset.

Method	mAP	Rank-1	Rank-5
Aligned-reID [3]	64.8%	81.2%	92.4%
PPbM-a [3]	74.1%	88.2%	96.4%
PPbM-b [3]	72.8%	89.4%	95.6%
MPFNet [22]	79.8%	95.4%	98.6%
Ours	78.7%	96.3%	98.9%

**Table 4:** Performing ablation experiments on the ATRW test dataset to demonstrate the effectiveness of the IFPM.

Method	mAP	Rank-1	Rank-5
ResNet50	74.4%	93.4%	98.4%
ResNet50 + FPN	66.7%	91.7%	97.6%
ResNet50 + IFPM (Ours)	76.1%	96.3%	98.9%

**Table 5:** Performing ablation experiments on the ATRW test dataset to demonstrate the effectiveness of the LAME.

Method	mAP	Rank-1	Rank-5
ResNet50	74.4%	93.4%	98.4%
ResNet50 + IFPM (Ours)	76.1%	96.3%	98.9%
ResNet50 + IFPM + LAEM (Ours)	78.7%	96.3%	98.9%

**Table 6:** Ablation experiments on ATRW test data set prove the progressiveness of CBAM module in the local branch.

Method	mAP	Rank-1	Rank-5
ResNet50	74.4%	93.4%	98.4%
ResNet50 + IFPM + SENet	76.1%	94.7%	98.4%
ResNet50 + IFPM + ECA	76.1%	95.4%	98.4%
ResNet50 + IFPM + LAEM (Ours)	78.7%	96.3%	98.9%

## Data Availability

The data presented in this study are available on request from the corresponding author.
